# PET-Derived Radiomics and Artificial Intelligence in Breast Cancer: A Systematic Review

**DOI:** 10.3390/ijms232113409

**Published:** 2022-11-02

**Authors:** Luca Urso, Luigi Manco, Angelo Castello, Laura Evangelista, Gabriele Guidi, Massimo Castellani, Luigia Florimonte, Corrado Cittanti, Alessandro Turra, Stefano Panareo

**Affiliations:** 1 Department of Translational Medicine, University of Ferrara, Via Aldo Moro 8, 44124 Ferrara, Italy; 2 Nuclear Medicine Unit, Oncological Medical and Specialist Department, University Hospital of Ferrara, 44124 Cona, Italy; 3 Medical Physics Unit, Azienda USL of Ferrara, 44124 Ferrara, Italy; 4 Medical Physics Unit, University Hospital of Ferrara, 44124 Cona, Italy; 5 Nuclear Medicine Unit, Fondazione IRCCS Ca’ Granda, Ospedale Maggiore Policlinico, 20122 Milan, Italy; 6 Department of Medicine DIMED, University of Padua, 35128 Padua, Italy; 7 Medical Physics Unit, University Hospital of Modena, 41125 Modena, Italy; 8 Nuclear Medicine Unit, Oncology and Haematology Department, University Hospital of Modena, 41125 Modena, Italy

**Keywords:** radiomics, artificial intelligence, AI, machine-learning, deep-learning, breast cancer, positron emission tomography, PET/CT

## Abstract

Breast cancer (BC) is a heterogeneous malignancy that still represents the second cause of cancer-related death among women worldwide. Due to the heterogeneity of BC, the correct identification of valuable biomarkers able to predict tumor biology and the best treatment approaches are still far from clear. Although molecular imaging with positron emission tomography/computed tomography (PET/CT) has improved the characterization of BC, these methods are not free from drawbacks. In recent years, radiomics and artificial intelligence (AI) have been playing an important role in the detection of several features normally unseen by the human eye in medical images. The present review provides a summary of the current status of radiomics and AI in different clinical settings of BC. A systematic search of PubMed, Web of Science and Scopus was conducted, including all articles published in English that explored radiomics and AI analyses of PET/CT images in BC. Several studies have demonstrated the potential role of such new features for the staging and prognosis as well as the assessment of biological characteristics. Radiomics and AI features appear to be promising in different clinical settings of BC, although larger prospective trials are needed to confirm and to standardize this evidence.

## 1. Introduction

Breast cancer (BC) represents the most common malignancy in terms of prevalence and the second cause of cancer-related death among women globally, with increasing incidence over the last decade [1]. BC is a heterogeneous tumor in terms of expression of several different receptors and genomic mutations. In particular, the receptor status classifies BC into four categories, i.e., Luminal A, Luminal B, Human epidermal growth factor receptor 2 (HER-2)+, and triple negative (TNBC), and also influences the choice of treatment options and the prediction of survival [2,3]. Nevertheless, tumor biology characterization is reliant on invasive procedures, such as biopsy sampling of a single lesion, which do not necessarily represent the whole tumor heterogeneity [4,5].

Hybrid imaging by [18F]F-Fluorodeoxyglucose positron emission tomography/computed tomography (FDG PET/CT), combining metabolic and morphological features, is now widely used for diagnosis, staging, assessment of treatment response and survival prediction of several malignancies, including BC [6,7,8]. Indeed, glucose metabolism reflects the biology of malignant cells and metabolic parameters, such as the maximum standardized uptake value (SUVmax), the mean standardized uptake value (SUVmean), the metabolic tumor volume (MTV) and the total lesion glycolysis (TLG), have been found to be associated with hormone receptors status and molecular subtypes in some studies [9,10,11,12]. Nonetheless, also semi-quantitative parameters derived from FDG PET/CT images have their own disadvantages; for example, SUVmax represents only the single hottest pixel, whereas MTV depends on the threshold-based method, and therefore, is not able to fully capture BC heterogeneity [13].

In the context of increasingly personalized medicine, the identification of reliable and non-invasive biomarkers able to predict tumor heterogeneity is fundamental for a patient’s treatment. Radiomics, defined as the process of identifying mineable variables hidden in the pixels of images and routinely not visualized by the human eye, is currently an emerging technique in the field of medical image analysis. Radiomics consists of high-throughput extraction, automated or not, of a large number of quantitative parameters from medical images, based on the hypothesis that such extracted features could be linked to genotypic and molecular characteristics of the tumor lesions. Its non-invasive nature and the possibility to study and to follow all lesions’ distributions over time, avoiding the requirement for repeated biopsies, are the undoubted advantages of radiomics [14,15,16]. In the setting of BC, radiomics data are available from several studies conducted with different medical images, such as ultrasound, mammography, magnetic resonance imaging (MRI), and PET/CT [17,18,19,20].

Artificial intelligence (AI) is a branch of computer sciences [21], which includes machine learning (ML) and deep learning (DL) [22]. ML models are based on a training dataset that is first provided to develop their own logic for answering future questions. DL is the newest class of ML and has been found to be advantageous to other forms of ML [23]. The development of computer science algorithms, tools and applications relevant to medical imaging has rapidly increased in the last years [24,25]. Aktolun [26] in 2019 describes the potential and challenges of radiomics and AI in nuclear medicine.

The aim of our systematic review is to summarize the current role of PET radiomics in BC, and to describe its potential application in clinical practice to assist physicians improve patient management.

## 2. Results

**Figure 1:**
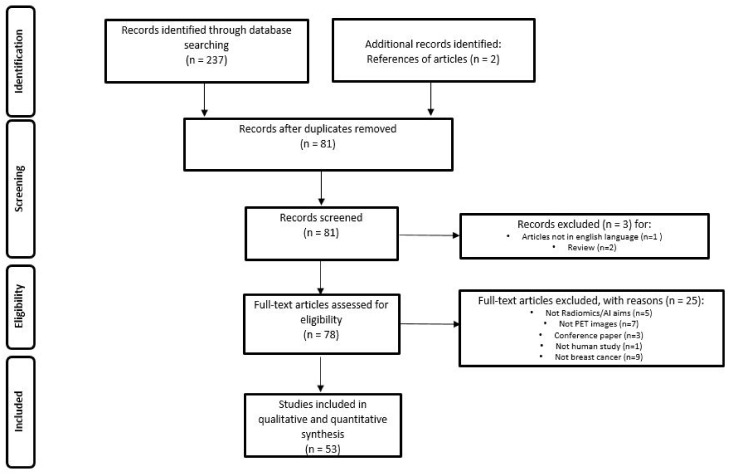
PRISMA Flowchart of study selection and inclusion in the systematic review.

The literature search identified a total of 239 studies, reduced to 81 after excluding duplicates, non-original articles, and papers relevant to other topics. The number of studies that met the inclusion criteria was 53. Figure 1 illustrates the PRISMA flowchart of the articles included in our systematic review. Among these, 43 (81.1%) studies were retrospective and 10 (18.9%) prospective. Radiomic data were extracted from PET/CT in 43 (81.1%) papers, PET/MRI in 4 (7.6%) and dedicated breast PET (dbPET) in 6 (11.3%) papers. The majority of the studies were performed using [18F]-fluorodeoxyglusoce ([18F]-FDG) (50 studies, 94.3%), but few experiences were reported using [18F]F-Fluorestradiol (18F-FES, 1 study, 1.9%) and [18F]F-Fluorothymidine (18F-FLT, 2 studies, 3.8%).

The papers were divided into six categories according to the clinical context explored and/or the type of study: diagnosis and biological characterization, neoadjuvant chemotherapy (NAC), staging and restaging, prognosis, dbPET, and technical papers. The number of papers included in each category is shown in Table 1. Some papers were included in more than one category, based on the discussed aim.

Overall, 47 out of 53 papers (88.7%) performed texture analysis, using several software packages, mostly open source (n = 26; 49.1%). Data mining was performed in 24 papers (45.3%) using ML and DL in 18 and 6 cases, respectively. In 20 out of 24 (83.3%) studies performing data mining, validation was also performed.

**Table 1:** Summary of general characteristics of studies.

Author	Year	Design	Clinical Context/Type of Study	Aim	RP	Scanner	N. Pts.
Liu et al. [27]	2021	R	Diagnosis and biological characterization	To predict molecular subtype classification of BC	FDG	PET/CT	273
Krajnc et al. [28]	2021	P	Diagnosis and biological characterization	To characterize BC	FDG	PET/CT	170
Ou et al. [29]	2020	R	Diagnosis and biological characterization	To differentiate BC from breast lymphoma	FDG	PET/CT	44
Ou et al. [30]	2019	R	Diagnosis and biological characterization	To differentiate BC from breast lymphoma	FDG	PET/CT	44
Acar et al. [31]	2019	R	Diagnosis and biological characterization	To predict immunochemistry and prognosis in BC	FDG	PET/CT	72
Antunovic L et al. [12]	2017	R	Diagnosis and biological characterization	To predict Immunochemistry and subtypes in BC	FDG	PET/CT	43
Groheux D et al. [32]	2015	P	Diagnosis and biological characterization	To predict immunochemistry, subtypes and pCR in BC	FDG	PET/CT	146
Yoon HJ et al. [33]	2015	R	Diagnosis and biological characterization	To predict invasive components in breast ductal carcinoma in situ	FDG	PET/CT and MRI	65
Soussan M et al. [34]	2014	R	Diagnosis and biological characterization	To predict BC prognosis	FDG	PET/CT	54
Umutlu et al. [35]	2022	R	NAC	To predict response after NAC	FDG	PET/MRI	73
Fantini et al. [36]	2021	P	NAC	To predict response after NAC	FLT	PET/CT	15
Choi et al. [37]	2020	R	NAC	To predict response after NAC	FDG	PET/CT and PET/MRI	56
Li et al. [38]	2020	R	NAC	To predict response after NAC	FDG	PET/CT	100
Antunovic et al. [39]	2019	R	NAC	To predict response after NAC	FDG	PET/CT	291
Lee H et al. [40]	2019	R	NAC	To predict response after NAC	FDG	PET/CT	435
Willaime J M Y et al. [41]	2013	R	NAC	To predict response after NAC	FLT	PET	15
Cheng et al. [42]	2022	R	NAC	To predict ALN mts	FDG	PET/CT	290
Eifer et al. [43]	2022	R	Staging/re-staging	To differentiate between ALN mts and inflammation	FDG	PET/CT	99
Chen et al. [44]	2022	R	Staging/re-staging	To detect occult ALN mts in cN0	FDG	PET/CT	180
Araz et al. [45]	2022	R	Staging/re-staging	To predict HR positivity	FDG	PET/CT	153
Moreau et al. [46]	2022	P	Staging/re-staging	To detect BC mts and to determine treatment response in metastatic BC	FDG	PET/CT	60
Lee et al. [47]	2021	R	Staging/re-staging	To predict ALN mts	FDG	PET/CT	326
Li et al. [48]	2021	R	Staging/re-staging	To predict ALN mts	FDG	PET/CT	407
Song et al. [49]	2021	R	Staging/re-staging	To predict ALN mts	FDG	PET/CT	100
Schiano et al. [50]	2019	R	Staging/re-staging	To detect early mts	FDG	PET/MRI	217
Jo et al. [51]	2022	R	Prognosis	To evaluate RFS	FDG	PET/CT	124
Bouron et al. [52]	2022	P	Prognosis	To predict prognosis in TNBC	FDG	PET/CT	111
Weber et al. [53]	2021	R	Prognosis	To predict BC prognosis	FDG	PET/CT	50
Chang et al. [54]	2019	R	Prognosis	To predict BC prognosis	FDG	PET/CT	35
Groheux et al. [55]	2017	R	Prognosis	To predict immunochemistry and prognosis in BC	FDG	PET/CT	143
Satoh et al. [56]	2022	R	dbPET	To predict BC	FDG	PET/CT and dbPET	284
Satoh et al. [57]	2020	R	dbPET	To characterize BC	FDG	PET/CT and dbPET	44
Hathi et al. [58]	2020	R	dbPET	To characterize BC	FDG	PET/CT and dbPET	10
Moscoso A et al. [59]	2018	R	dbPET	To predict immunochemistry and subtypes in BC	FDG	dbPET	127
Satoh et al. [60]	2020	R	dbPET	To predict BC	FDG	dbPET	105
Cheng L et al. [61]	2017	R	dbPET	To predict response after NAC	FDG	PET/CT and dbPET	61
Castaldo et al. [62]	2022	P	Technical papers	Framework of analysis to generate a combined radiomic signature in BC	FDG	PET/MRI	36
Takahashi et al. [63]	2022	R	Technical papers	Deep learning to improve diagnostic accuracy in BC	FDG	PET/CT	500
Aide et al. [64]	2018	P	Technical papers	PET/CT acquisition protocol optimization to improve BC diagnosis	FDG	PET/CT	47
Boughdad S et al. [65]	2018	R	Technical papers	Robustness of textural features in BC	FDG	PET/CT	552
Orlhac F et al. [66]	2018	R	Technical papers	Harmonization method for multicenter radiomic study	FDG	PET	63
Yang Z et al. [67]	2017	R	Technical papers	Assessment of estrogen receptor from intratumoral heterogeneity	FES	PET/CT	46
Orlhac F et al. [68]	2017	R	Technical papers	Robustness of textural features in BC	FDG	PET/CT	54
Hatt M et al. [69]	2015	R	Technical papers	Textural features to quantify intratumoral BC heterogeneity	FDG	PET/CT	555
Orlhac F et al. [70]	2014	P	Technical papers	Robustness of textural features in BC	FDG	PET/CT	106
Chen et al. [71]	2022	R	Diagnosis and biological characterization/Staging re-staging	To predict HER2 expression in BC	FDG	PET/CT	271
Umutlu et al. [72]	2021	R	Diagnosis and biological characterization/NAC	Breast cancer phenotyping and tumor decoding	FDG	PET/MRI	124
Molina Garcia et.al [73]	2018	P	Diagnosis and biological characterization/NAC	To predict BC prognosis	FDG	PET/CT	68
Lemarignier et al. [74]	2017	R	Diagnosis and biological characterization/NAC	To characterize BC (T stage, stage and histology)	FDG	PET/CT	171
Ha et al. [75]	2017	R	Diagnosis and biological characterization/NAC/Prognosis	To predict immunochemistry, response after NAC and prognosis in BC	FDG	PET/CT	73
Aide et al. [76]	2021	P	Diagnosis and biological characterization/Prognosis	To predict immunochemistry and prognosis in BC	FDG	PET/CT	38
Huang et al. [77]	2018	R	Diagnosis and Biological Characterization/Prognosis	To predict immunochemistry and prognosis in BC	FDG	PET/CT	113
Yoon et al. [78]	2019	R	NAC/Prognosis	To predict response after NAC	FDG	PET/CT and MRI	83

ALN: axillary lymph nodes; BC: breast cancer; BSGI: breast-specific gamma imaging; dbPET: dedicated breast PET; FDG: [18F]F-Fluorodeoxyglucose; FES: [18F]F-Fluorestradiol; FLT: [18F]F-Fluorothymidine; HR: hormone receptors; HER-2: Human epidermal growth factor receptor 2; MTS: metastases; NAC: neoadjuvant chemotherapy; pCR: pathological complete response; P: prospective; R: retrospective; RFS: relapse free survival; RP: radiopharmaceutical; TNBC: TN breast cancer.

### 2.1. Diagnosis and Biological Characterization

The wide reach of screening programs allows earlier detection of BC in the population, significantly improving patients’ outcomes [79]. However, BC screening has some limitations and improving radiological performances in this subset is an unmet need of oncology [80,81,82,83]. Indeed, after detecting a lesion with screening programs, a biopsy is usually performed, with a certain number of cases hesitating in benign or uncertain findings. Therefore, several authors have tried to solve this issue by applying radiomics to many imaging modalities, including PET/CT/MRI, to obtain “free” information on newly diagnosed breast lesions from already available imaging data. Krajnc et al. [28] found a high performance of holomic models in BC detection (80% sensitivity, 78% specificity, 80% accuracy, 0.81 area under the curve (AUC)) and TNBC tumor identification (85% sensitivity, 78% specificity, 82% accuracy, 0.82 AUC). For the same purpose, an intermediate result was obtained with the SUVmax model (AUC 0.76 in cancer detection and 0.70 in the prediction of TNBC subtype). Conversely, holomic models demonstrated only low performance for determining receptor status and luminal A/B subtype (0.46–0.68 AUC). Moreover, Lemarignier et al. [74] reported that all the texture features extracted from FDG PET demonstrated significant correlations with tumor size (T2 vs. T3), the American Joint Committee on Cancer stage (stage II vs. stage III) and the histological type (invasive ductal carcinoma vs. invasive lobular carcinoma).

Yoon et al. [33] performed a texture-based analysis of intratumoral metabolic heterogeneity aiming to reveal the presence of tumoral invasive components in a retrospective analysis of 65 patients undergoing FDG PET/CT for ductal carcinoma in situ (DCIS). The authors reported a lower AUC of cumulative SUV histograms (AUC-CSH), a parameter reflecting higher intratumoral metabolic heterogeneity, was associated with an underestimation of invasive components. Thus, sentinel lymph node biopsy should be considered in patients affected by DCIS with low AUC-CSH.

Ou and colleagues [29,30] investigated the ability of FDG PET/CT radiomic features to discriminate BC from breast lymphoma using a ML approach. PETa (based on clinical, SUV and radiomic features from PET images) and CTa (clinical and radiomic features from CT images) models showed the best ability to discriminate between two different breast malignancies, both in training and in validation groups (AUCs of 0.867 and 0.806 for PETa model, AUCs of 0.891 and 0.759 for CTa model, respectively). Moreover, SUV metrics (particularly SUVmax) extracted from FDG PET/CT images showed potential in the differentiation between breast lymphoma and carcinoma as well as for the differentiation of the different subtypes of lymphoma [30].

Another important application of radiomics is in the biological characterization of BC. Indeed, different BC subtypes present very different behaviours in terms of incidence, clinical-pathological features, disease natural history and prognosis [84]. Therefore, several authors applied radiomic features to predict the biologic characterization of BC [12,73,77]. Liu et al. [27] compared conventional PET parameters (SUVmax, SUVmean, SUVpeak, MTV, TLG) and PET-derived radiomic features in the prediction of molecular subtype classification of BC. As a result, PET-derived radiomic features outperformed every individual conventional PET parameter, including luminal vs. non-luminal (AUC = 0.913 vs. AUC = 0.725), HER-2+ vs. HER-2– (AUC = 0.912 vs. AUC = 0.820), and TNBC vs. non-TNBC classification (AUC = 0.968 vs. AUC = 0.901). Similarly, Umutlu et al. [72], in a cohort of 124 patients undergoing FDG PET/MRI, extracted radiomic features from both types of images (PET and MRI). The authors found that texture features extracted from MRI images had the best performance in differentiating luminal A from luminal B cancers (AUC = 0.98; accuracy = 97.3). Moreover, PET-derived radiomic features provided the best accuracy in the grading determination (AUC = 0.71), while both PET and MRI-derived features could predict hormone receptor status (AUC = 0.87 and 0.88 for estrogen receptor (ER) and progesterone receptor (PR), respectively), tumoral proliferation index, expressed in Ki-67 (AUC = 0.997), and lymph nodes and distant metastatic disease (AUC = 0.81 and 0.99, respectively). A similar result was reported by Aide et al. [76]. In a cohort of 38 luminal non-metastatic BC, the authors extracted some radiomic features able to correlate the heterogeneity of metabolic activity on FDG PET with that of ER and PR expressions. Conversely, in 153 patients who underwent preoperative FDG PET/CT, Araz et al. [45] found that only SUVmax, SUVmean, and SUVpeak were significantly higher in HR negative patients, whereas none of the radiomics features were predictors for HR status. This result is consistent with that published by other papers [74], in particular Ha et al. [75], who reported that three tumor clusters obtained by unsupervised clustering based on FDG PET-related texture features were not associated with ER, PR, or HER-2 status, but only with ki-67 index. Likewise, Groheux et al. [32] did not find radiomic features correlated with clinical and histopathological characteristics or with BC subgroups in a cohort of 171 patients with stage II-III BC. Acar et al. [31] reported an intermediate result between the papers described above. In this work, both conventional and radiomic parameters extracted from FDG PET correlated with ER expression, but only conventional PET metrics were able to predict Ki-67 index and the status of PR and HER-2. Chen et al. [71] investigated the ability of ML, based on FDG PET, to predict HER-2 status in BC patients. The best results were obtained by the XGBoost model based on PET/CTmean or PET/CTconcat radiomic fusion features. Finally, Soussan et al. [34] reported that the best performance in identifying TNBC was obtained by combining SUVmax and High-Gray-level Run Emphasis (HGRE), a textural index extracted from delineated tumor volume on FDG PET/CT (AUC = 0.83).

### 2.2. Neoadjuvant Chemotherapy

The treatment of locally advanced BC (LABC) usually differs from that of early BC (EBC) as in LABC, surgery is usually preceded by neoadjuvant chemotherapy (NAC) [85]. However, NAC indications are have been widely debated in the literature after recent evidence suggested consideration of NAC in TNBC and HER-2-positive BC, regardless of disease extension [86]. The literature underlines the essential value of pathological complete response (pCR) after NAC, which is significantly associated with prolonged disease free survival (DFS) and overall survival (OS) [87,88,89]. In this context, several authors extracted radiomic features to predict pCR from baseline FDG PET/CT. Umutlu et al. [35] recently assessed the potential role of baseline multiparametric FDG PET/MRI-based radiomics to predict pCR after NAC in 73 female patients with newly diagnosed therapy-naïve BC. The combination of all MRI sequences and PET data showed the best results in terms of AUC and negative predictive value (NPV) (0.8 and 79.5%, respectively). Moreover, in a subgroup of HR+/HER-2− patients, the best AUC (0.94) for predicting pCR was obtained by combining all the MRI and PET data. This result is consistent with the previous works by Antunovic et al. [39], Lee et al. [40], Yoon et al. [78] and Ha et al. [75]. Similarly, Li et al. [38] found some baseline FDG PET/CT derived radiomic features able to predict efficacy prior to NAC (prediction accuracy (PA) = 0.857; AUC = 0.844 on the training split set and PA = 0.767; AUC = 0.722 on the independent validation set) in a retrospective analysis of 100 BC patients. Interestingly, incorporating age in the analysis improved PA to 0.857 (AUC = 0.958) and 0.8 (AUC = 0.73) for the split set and independent validation set, respectively, outperforming the clinical prediction model. Similarly, Molina-Garcia et al. [73] reported the usefulness of textural variables obtained from baseline FDG PET/CT before NAC for predicting OS and DFS. Interestingly, radiomic-derived parameters were equally as strong at predicting patient outcomes as PET at the diagnosis stage.

Choi and colleagues [37] in a further step, introduced a breast PET/MRI image deep learning model (convolutional neural network—CNN) and compared it with the conventional parameters. They found that the application of CNN could improve the AUC of conventional parameters, except for baseline diffusion MRI images.

Conversely, in the work by Lemarignier et al. [74] and Cheng et al. [61], radiomic features could not predict pCR. However, the modifications in conventional and radiomic PET features, evaluated between baseline and after two cycles of NAC, resulted in increased predictive strength for pCR.

Investigating the clinical value of [18F]F-fluoroestradiol (FES) in the assessment of the ER status and its intratumoral heterogeneity expression in BC patients was the main aim of the work published by Yang et al. [67]. These authors found a good correlation between FES, FDG uptake (SUVmax and SUVmean), and pathological features (ER, PR, HER-2, Ki67%, and tumor size). Furthermore, they suggest the use of SUVmean instead of SUVmax because it provided a slightly better correlation between quantitative tumor FES uptake and hormone receptor expression (ER, PR) and HER-2 amplification.

Finally, Fantini and colleagues [36] extracted radiomic advanced textural features from [18F]F-FLT (FLT) PET/CT and explored their accuracy in the prediction of response to NAC in a cohort of 15 patients with LABC. A combination of SUVmax and textural feature index IVH_VolumeIntFract_90 was identified as the best combination to classify PET response. Moreover, the combination of PET response, ID range, and ID_Coefficient of Variation was able to classify pathological response to NAC. A similar result was obtained by Willaime et al. [41], who showed a correlation between FLT PET/CT derived radiomic features and both partial pathological response and pCR after NAC in 14 BC patients.

### 2.3. Staging and Restaging

FDG PET/CT is an imaging modality widely used in oncology to assess the glycolytic metabolism and is based on the so-called “Warburg effect” [90]. Specifically, malignant cells have an increased glucose metabolism in comparison with normal tissues, and this metabolic change can be easily detected by FDG PET/CT in numerous malignancies, including BC [91]. In particular, in patients with BC, axillary lymph node (ALN) metastasis is one of the most significant clinical factors, dictating the treatment strategy and predicting survival [92]. For this reason, in the last years, several papers have focused on the potential diagnostic role of PET radiomics for predicting ALN metastasis [43,44,47,48,49]. Li et al. [48] constructed an AI-assisted diagnosis system using deep-learning technology to improve clinicians’ diagnostic accuracy in the identification of ALN metastasis. They analyzed 404 BC patients who underwent FDG PET/CT before surgery. The AI model did not outperform the clinicians’ image analysis, but the diagnostic accuracies were considerably improved when combining both evaluations. Indeed, the two clinicians’ sensitivities of 59.8% and 57.4% increased to 68.6% and 64.2%, respectively, whereas the clinicians’ specificities of 99.0% and 99.5% remained unchanged. The authors concluded by suggesting a possible assistance role for AI in assisting clinicians in ambiguous cases.

On the other hand, Song and colleagues [49] proposed a ML-based radiomic model developed analysing FDG PET/CT with the aim of predicting ALN metastasis in a cohort of 100 patients with invasive ductal BC. The model showed excellent results (90.9%, 71.4%, and 80% for sensitivity, specificity, and accuracy, respectively), which suggest it as a promising tool for the preoperative detection of ALN metastasis.

Regardless of negative preoperative investigations (including ultrasound imaging, PET/CT, or fine-needle aspiration), some cN0 patients develop metastases. As a consequence, sentinel lymph node biopsy (SLNB) and axillary lymph node dissection (ALND) are frequently performed, although they are invasive procedures not free from complications [93,94]. Therefore, finding a non-invasive tool able to detect occult ALN metastases in cN0 patients would be very useful. In this subset of patients, Chen et al. [44] identified 14 FDG PET/CT-derived radiomics features able to perceive ALN metastasis. Then, random forest (RF), support vector machine (SVM), stochastic gradient descent (SGD), and k-nearest neighbour (KNN) were used to build the prediction models. Among the four models, RF showed the highest accuracy (mean AUC 81.2%, *p* < 0.001) and could potentially help the clinicians in determining ALN status in patients with cN0.

Furthermore, Lee et al. [47] investigated the role of textural parameters, extracted from peritumoral breast adipose tissue, on pre-operative FDG PET/CT in predicting ALN metastasis in 326 BC patients. Among 38 features extracted, the highest AUC value (0.830) was shown by grey-level co-occurrence matrix (GLCM) entropy, which outperformed visual analysis (0.739, *p* < 0.05) and was comparable to LN SUVmax (0.793, *p* < 0.05). Interestingly, GLCM entropy could also predict ALN metastasis in patients with negative findings on visual analysis (AUC: 0.759). This study highlights the importance of tumor microenvironment, such as adipose tissue, in the progression and metastatic spread of BC.

In the era of COVID-19 mRNA vaccination, a new challenge faced by clinicians is the correct differentiation between metastatic and reactive axillary LN. In their retrospective study, Eifer et al. [43] aimed to differentiate between metastatic axillary lymphadenopathy in BC patients and reactive inflammatory LN in those who received anti-COVID-19 vaccine using a radiomics and a ML approach. According to the RF and KNN models, combined PET/CT features had the highest AUC values for differentiating between axillary metastasis and inflammation post-vaccination, followed by CT and PET features. In particular, the first-order, GLRLM, and GLDM features were those with AUC values above 0.9. Based on these results, the authors suggest a potential application in discriminating between benign and malignant LN.

Although most of the studies have been based on PET/CT, Schiano et al. [50] have combined radiomics parameters from hybrid FDG PET/MRI with the expression level of the transcriptional factor Yin Yang 1 (YY1) for the detection of early metastases. YY1 level was significantly overexpressed in the ER+/PR+/HER-2- subtype of BC patients with synchronous metastasis at staging compared with metachronous metastasis and healthy subjects (*p* < 0.001), and it correlated significantly with SUVmax (r = 0.48). Hence, the combination of functional FDG PET/MRI parameters and molecular determination of YY1 could represent a novel integrated approach to predict synchronous metastatic disease with more accuracy than FDG PET/MRI alone.

In the last decades, several criteria, both morphological and metabolic (e.g., RECIST and PERCIST), have been proposed to assess treatment response in oncology. Nevertheless, manual segmentation of all lesions is time consuming in clinical practice, especially in patients with multiple metastases. For this reason, Moreau and colleagues [46] trained two deep-learning models in order to automatically segment BC metastatic lesions on the baseline and follow-up FDG PET/CT of 60 patients. The authors assessed four imaging biomarkers, i.e., SULpeak, TLG, PET Bone Index, and PET Liver Index, with SULpeak identified as the best biomarker to assess patients’ response (sensitivity 87%, specificity 87%), representing a promising tool for automatic segmentation of metastatc BC lesions.

### 2.4. Prognosis

Several variables concur in defining BC prognosis, including clinical-pathological features and treatment selection [9,95]. Moreover, new insights in imaging analysis demonstrated an incremental value in stratifying the prognosis of BC patients [81]. Among imaging modalities, several papers have reported the prognostic meaning of FDG PET/CT [8,96]. Bouron et al. [52] aimed to identify the association among metabolic, volumetric and textural parameters extracted from FDG PET/CT at diagnosis and clinical outcomes, expressed by DFS and OS, in 111 TNBC patients. Five metabolic and volumetric parameters (i.e., SUVmax, SUVmean, SUVpeak, MTV, and TLG), and six textural features (i.e., entropy, homogeneity, Short-Run Emphasis, Long-Run Emphasis, Low-Gray-level Zone Emphasis, and High-Gray-level Zone Emphasis) derived from the primary tumor were analysed. While in the univariate analysis, high TLG, MTV and entropy of the primary tumor were associated with DFS and OS, in the multivariate analysis only MTV of the primary tumor, with a threshold value of 9.3, correlated with a shorter OS. Similarly, two more studies reported that, on multivariate analysis, primary tumor MTV was an independent predictor of relapse free survival (RFS) [51] and event free survival (EFS) [55], respectively, whereas textural analysis of PET images did not show any added value. In another study with similar aims, only MTVwb was an independent predictor for shorter progression free survival (PFS) in 35 patients with newly diagnosed invasive ductal BC (HR: 8.29, 95% CI: 2.17–31.64, *p* = 0.0020) [54]. Moreover, a higher clinical stage was found to be an independent prognostic factor for OS.

A prognostic significance for FDG PET radiomic features was found by Aide et al. [76]. Skewness_ER was identified as a predictor of 8y-EFS using the univariable Kaplan–Meier method, although this was not confirmed by multivariate analysis. Moreover, Yoon et al. [78] found that only high-intensity zone emphasis was a significant predictor of recurrence (*p* = 0.027) in a cohort of 83 patients with LABC who underwent FDG PET at diagnosis.

Two papers applied AI on FDG PET to obtain prognostic data. In the paper published by Huang et al. [77], the three tumor clusters identified with an unsupervised clustering of FDG PET and MRI-derived parameters showed a significant correlation not only with tumor molecular subtypes and immunohistochemistry, but also with relapse free survival (RFS). This is consistent with the results of Ha et al. [75]. Furthermore, Weber et al. [53] evaluated the accuracy of a neural network, trained for lymphoma and lung cancer, in the correct detection and segmentation of pathological uptakes in patients with advanced BC. Surprisingly, the authors report a high correlation between AI-derived and manually segmented MTV (R2 = 0.91; *p* < 0.001). Moreover, in multivariate analysis, AI-derived MTV (both whole body and organ-wise) resulted a predictor of OS.

### 2.5. dbPET

dbPET consists of high resolution molecular imaging acquired on hanging uncompressed breast, using a high resolution full-ring breast-dedicated tomograph [97]. The first experience using dbPET was published by Moliner et al. [98] in 2010. This imaging modality provides a very high detection rate, thanks to its 1.5–2.0 mm spatial resolution [99]. In a few studies, textural features were extracted to make a direct comparison between dbPET and whole-body PET [57,58,59]. Satoh et al. [57], in a retrospective study of 44 patients, compared the two tomographs in classifying tumor characteristics of BC, obtaining similar results for both dbPET and whole-body PET/CT. Conversely, Moscoso et al. [59], demonstrated strong correlations between FDG dbPET-derived radiomic features and both immunohistochemistry and molecular subtypes of BC, stronger than those obtained by whole-body PET. Hathi et al. [58] characterized similarities and differences in the uptake of FDG between bilateral dbPET and wbPET in a cohort of ten patients with biopsy-confirmed LABC before starting NAC. FDG uptake measurements and 20 radiomic features related to morphology, tumor intensity, and texture were calculated and compared to predict the response to NAC. dbPET-derived features outperformed wbPET ones when using SULpeak (five times increased in comparison with wbPET) and spatial heterogeneity features. The authors conclude that dbPET could be useful for prediction of primary tumor response to NAC.

Analysing dbPET images, Satoh et al. [60] developed a ML model with SVM including quantitative parameters that was able to detect early BC using dbPET. They found that SVM outperformed visual assessment for this purpose (0.77 vs. 0.89, 0.57 vs. 0.94, 0.77 vs. 0.77 and 0.71 vs. 0.85, for AUC, sensitivity, specificity, and accuracy, respectively). Cheng et al. [42] aimed to develop a ML model combining dbPET features and clinical variables to predict pathological involvement of ALN in 420 early-stage BC. The AUC of the integrated model, which included six clinical-pathological factors and five dbPET radiomics parameters, was 0.94 in the training set (n = 203) and 0.93 in the validation set (n = 87) (*p* < 0.05 in both cases). Moreover, in the clinical N0 subgroup, NPV and PPV were 96.9% and 92.7%, respectively. The study highlights the potential positive impact of ML for improving true negative and true positive detection of ALN.

More recently, Satoh et al. [56] attempted to determine the best DL model to predict BC. The model was trained with 458 breasts (including 109 breast and 349 non-breast cancers) and tested with 160 breasts, comprehending 43 cancers and 117 non-breast cancers. The deep learning model showed 93% for both sensitivity and specificity, compared with 77–89% and 79–100% obtained from two expert radiologists. In addition, the diagnostic performance of the model (AUC = 0.937) was not significantly different from that of the experts (AUC = 0.983, *p* = 0.095; AUC = 0.941, *p* = 0.907).

### 2.6. Techincal Papers

The lack of standardization of features calculation and methodology hinders comparisons of the results of radiomic studies in the literature [68,100]. After features extraction, the reproducibility of each features, robustness and sensitivity should be investigated [101]. To allow for texture index value interpretation, Orlhac et al. [68] investigated the changes in value of six texture indices computed from simulated and real patient data. Variability in texture index values as a function of voxel size (variations up to 85.8% for the most homogeneous sphere model) and edge effects (variations up to 29%) was demonstrated.

Boughdad et al. [65] found significant SUVs and textural features differences as a function of age in normal breast tissue and in BC radiomic phenotype with triple-negative tumors being the most affected. Their results suggest that age should be considered as a covariable in radiomic models.

In order to clarify the relationship between texture features and conventional indices (SUV, MTV, TLG), Orlhac et al. [70] studied 31 different TFs in 3 different tumor types. They reported that only 17 of 31 texture indices were robust with respect to the tumor segmentation method. Additionally, they proposed that a resampling formula with at least 32 gray levels should be used to preserve the relationship between textural features and SUV. Moreover, Hatt et al. [69] demonstrated the correlation (Spearman rank correlation rs = 0.74) between tumor heterogeneity (entropy) and metabolic tumor volume in a multi-cancer site BC patient cohort.

A further complexity occurs in multicenter studies. It is necessary to remove the center effect (i.e., scanner, acquisition protocol) while preserving patient-specific effects. Orlhac et al. [66] proposed a post-reconstruction harmonization method efficient at removing multicenter effects for textural features and SUVs. After harmonization, none of the nine features, extracted form healthy liver tissue ROI in BC patients, significantly differed between the two departments (*p* > 0.1).

The influence of acquisition protocol and reconstruction setting on TFs was investigated by Aide et al. [64]. A prediction model for tumor classification was built using a random forests method. Matrix size and PSF modelling appeared to improve discrimination between immunohistochemical subtypes (luminal versus non-luminal) in breast cancer.

Recently, some authors have developed AI models in order to improve prognosis [62] and increase diagnostic accuracy [63] in BC patients. Castaldo et al. [62], in a pilot study, evaluated different normalization methods on primary component analysis (PCA), both within-subject and between-subjects, in order to generate a combined radiomic signature for a more precise breast cancer prognosis, helping clinicians to achieve improved therapeutic decision-making and make progress towards ever more personalized medicine. The results were compared and validated on twenty-seven patients to investigate the tumor grade, Ki-67 index, and molecular cancer subtypes using classification methods (LogitBoost, random forest, and linear discriminant analysis).

To increase the diagnostic accuracy of PET/CT, deep learning models using images derived from four different degrees (i.e., 0°, 30°, 60°, and 90°) of PET maximum-intensity projection (MIP) were developed by Takahashi et al. [63]. The models were trained with 400 images (200 cancers and 200 non-cancers) and tested on 50 breast and 50 non-breast cancers. The promising sensitivity (80% to 98%) and specificity (76% to 92%) obtained in the different models, suggest that a deep learning model may be able to assist radiologists in their diagnostic work in the future.

### 2.7. Radiomics Quality Assessment

**Figure 2:**
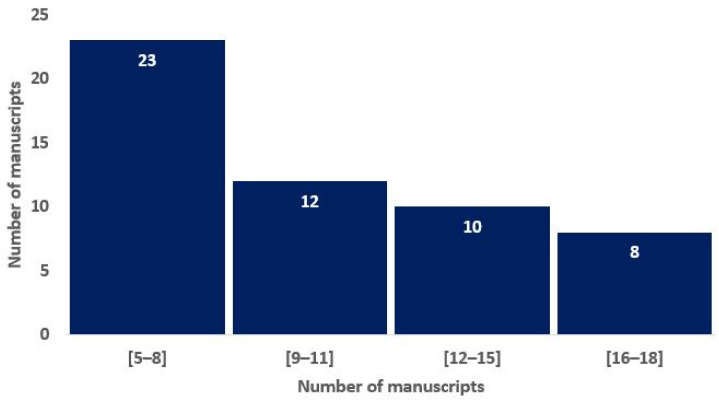
Histograms of average RQS scores according to the blinded analysis of two authors, based on the previous study of Lambin and colleagues [16].

To assess the overall quality of the considered radiomics studies, we adopted the RQS metric. All the considered studies had an RQS between 5 (13.89%) and 22 (61.11%). The distribution of the RQS scores of the studies in Figure 2 shows that most of the studies are non-compliant with the best-practice procedures. Nevertheless, 15% of the studies have achieved a score that is representative of a study that highly satisfies the research criteria in the radiomics area.

## 3. Materials and Methods

### 3.1. Literature Search Strategy

A search on the most relevant databases and online sources (Pubmed/Medline, Web of Science, Scopus) was performed running the following query string: “(PET OR Positron Emission Tomography OR PET/CT or PET/MRI) AND (Breast OR Breast Cancer) AND (Radiomics OR Texture OR Texture Analysis OR Machine Learning OR Deep Learning OR Artificial Intelligence OR AI) NOT REVIEW”. English-language original articles published before 15 June 2022 were considered.

### 3.2. Study Selection

Titles and abstracts were independently reviewed by three authors (L.U.; L.M.; and A.C.) to evaluate study inclusion. Full articles were retrieved when the abstract was considered relevant. Inclusion criteria applied during selection were as follows: (a) articles concerning BC; and (b) articles on texture analysis derived from PET/CT, PET/MRI and/or computer science applications. The following papers were considered ineligible: (a) review articles; (b) articles not in the English language; and (c) studies not within the field of interest (i.e., not radiomics/AI aims, not PET images, conference papers, not human studies, and not breast cancer). The data were summarized in a database with the following fields: first author, journal, year, title, exclusion/issues, imaging modality, computer science area, number of patients, training set size, test set size, validation set size, and the setting/purpose of the study (diagnosis and biological characterization, NAC, staging and restaging, prognosis, dbPET and technical papers) for the subsequent data analysis.

For each study, the radiomic analysis was assessed based on the radiomic quality score (RQS) [16]. For a robust calculation, RQS was blindly computed by two of the authors (L.U.and L.M.) and discrepancies were discussed to reach a consensus.

## 4. Discussion and Conclusions

This systematic review provides a state-of-the-art picture of the application of radiomic features and AI on FDG PET in BC. Due to its high prevalence, BC is a high impact neoplasm. Therefore, the application of radiomics and AI on this type of malignancy can have a very high relevance in terms of precision medicine, patient management and prognosis. However, although much work has been conducted in the last five years, further research is required before these approaches can be implemented in daily clinical practice. Promising results have been obtained in the characterization of the primary tumor characteristics, in particular, the molecular subtypes, although these have not been confirmed by all the studies analyzed. If validated in larger studies, or even through the use of big data, radiomics could provide an additional tool to further explore BC characteristics, alongside what breast biopsy already offers. In this context, the papers comparing dbPET and wbPET suggest the superiority of dbPET-derived radiomic features. Nevertheless, dbPET does not allow exploration of eventual metastases at a distance.

The application of PET radiomics for the prognostic stratification of BC has provided disappointing results to date, with quantitative parameters, particularly MTV, still appearing as the most reliable for this purpose [9]. Currently, the most interesting scenarios for PET radiomic application in BC appear to be the evaluation of ALN status and the prediction of pCR after NAC. The early identification of ALN metastasis has a large impact on a BC patient’s prognosis, as well as on the selection of the invasiveness of the surgical procedure performed (SLNB vs. ALND) [102]. Similarly, identifying patients who will not reach pCR after NAC is essential for offering a second-line therapy in patients requiring it [86]. However, PET radiomic studies are still very inhomogeneous and lack the reproducibility required for introduction into daily clinical practice, as already suggested by previous analyses [20,81,103]. In this context, considering the overview in Table 2 and Table 3, a trend for a rudimentary standardization seems to have started. The newest studies in the top half of the table at least report substantial information regarding the methods used for radiomic analysis, whereas the older studies at the bottom of the table lack methodological data. This might be considered a first step towards the use of solid, recognized radiomic analysis systems, which we hope will allow the widespread use of AI for selected applications in the near future.

Finally, some settings of BC still remain almost unexplored with radiomic analysis. In particular, the prediction of metastasis at distance at baseline PET imaging was performed by only one paper [50], but with encouraging results. Similarly, radiomic-assisted therapy response assessment was only explored by one study [46], which introduced an interesting automatic segmentation of BC lesions using DL. We encourage researchers to investigate the potentialities of radiomic analysis and AI also on these clinical settings of BC.

**Table 2:** Summary of studies’ radiomic features analyses (from newest to older).

Author	TA	FTs n.	FTs Types	Sw TA	Sw Class	Selected FTs	Statistical Test	RQS (%) [16]
Chen et al. [44]	Yes	3124	First-order, GLCM, GLRLM, GLDM, NGTDM, GLSZM, shape	3D slicer	OS	14	t-test, LASSO	15 (41.67%)
Umutlu et al. [35]	Yes	101	First-order, GLCM, GLRLM, NGLDM, NGTDM, GLSZM	ITK-SNAP	OS	6	elastic net combining Lasso and ridge regression	13 (36.11%)
Eifer et al. [43]	Yes	110	First-order, GLCM, GLRLM, GLDM, NGTDM, GLSZM, shape	Python Software	OS	18	t-test, Chi square	12 (33.33%)
Jo et al. [51]	Yes	7	First-order	Lifex	OS	7	ROC, Cox proportional-hazards model	10 (27.78%)
Cheng et al. [42]	Yes	851	First-order, GLCM, GLRLM, GLDM, NGTDM, GLSZM, Shape, Wavelat	3D slicer	OS	34	LASSO	13 (36.11%)
Castaldo et al. [62]	Yes	74	First- and second-order	PMOD	C	7	Spearman correlation, PCA	18 (50.00%)
Bouron et al. [52]	Yes	6	Homogeneity, entropy, SRE, LRE, LGZE, HGZE	DOSIsoft	C	6	PCA, ROC, log-rank test	16 (44.44%)
Araz et al. [45]	Yes	42	First- and higher-order	Lifex	OS	2	binary logistic regression analysis	7 (19.44%)
Satoh et al. [56]	No	nd	nd	nd	nd	nd	ICC, ROC	8 (22.22%)
Takahashi et al. [63]	No	nd	nd	nd	nd	nd	ICC, ROC	5 (13.89%)
Moreau et al. [46]	No	nd	nd	nd	nd	nd	Wilcoxon, Kolmogorov–Smirnov, Spearman correlation	5 (13.89%)
Chen et al. [71]	Yes	2436	First-order, GLCM, GLRLM, GLDM, GLSZM, shape	3D slicer	OS	34	ICC, variance threshold, Mann–Whitney U test	12 (33.33%)
Lee et al. [47]	Yes	38	First-order, GLCM, GLRLM, GLZLM, NGLDM	Lifex	OS	38	t-test, Chi square, ROC	10 (27.78%)
Liu et al. [27]	Yes	1710	First-order, GLCM, GLRLM, NGLDM, NGTDM, GLSZM	Matlab	C	1710	Wilcoxon and LASSO	12 (33.33%)
Fantini et al. [36]	Yes	148	First- and higher-order, shape	Matlab	C	39	ICC, LASSO	19 (52.78%)
Umutlu et al. [72]	Yes	101	First-order, GLCM, GLRLM, NGLDM, NGTDM, GLSZM	CERR	C	6	Lasso regression	14 (38.89%)
Krajnc et al. [28]	Yes	121	First-order, GLCM, GLSZM, NGTDM, shape	MUW	OS	77	Pearson correlation	22 (61.11%)
Weber et al. [53]	Yes	nd	nd	nd	OS	nd	Wilcoxon, Mann–Whitney U test	5 (13.89%)
Aide et al. [76]	Yes	nd	First-order, GLCM, NGLDM	Lifex	OS	nd	Mann–Whitney, ROC	19 (52.78%)
Li et al. [48]	No	nd	nd	nd	OS	nd	nd	8 (22.22%)
Song et al. [49]	Yes	792	nd	3D slicer	OS	30	nd	7 (19.44%)
Satoh et al. [57]	Yes	38	GLCM, GLRLM, GLZSM, NGLDM	PTexture	OS	38	PCA	8 (22.22%)
Hathi et al. [58]	Yes	20	First-order, GLCM, NGTDM, shape	3D slicer	OS	20	Wilcoxon	7 (19.44%)
Choi et al. [37]	No	nd	nd	nd	OS	nd	Chi square, Mann–Whitney U	8 (22.22%)
Satoh et al. [60]	No	nd	nd	nd	nd	nd	Chi square, Fischer, Mann–Whitney U	8 (22.22%)
Li et al. [38]	Yes	104	First-order, GLCM, NGTDM, GLRLM, GLSZM, GLDLM, shape	3D slicer	OS	10	Wilcoxon	13 (36.11%)
Ou et al. [29]	Yes	55	First-order, GLCM, NGLDM, GLRLM, GLZLM, shape	LifeX	OS	19	LASSO	11 (30.56%)
Schiano et al. [50]	Yes	12	GLCM	PMOD	C	15	Spearman correlation, Wilcoxon	12 (33.33%)
Chang et al. [54]	Yes	15	GLCM	CGITA	OS	15	Spearman correlation, ROC	9 (25.00%)
Ou et al. [30]	Yes	12	First-order, GLCM, GLRLM, NGLDM, GLZLM, shape	LifeX	OS	6	ROC	7 (19.44%)
Acar et al. [31]	Yes	nd	First-order, GLCM, GLRLM, NGLDM, GLZLM, shape	LifeX	OS	nd	Mann–Whitney U and Kruskal–Wallis	7 (19.44%)
Antunovic et al. [39]	Yes	nd	First-, second- and higher-order	LifeX	OS	nd	ROC, LASSO	11 (30.56%)
Aide et al. [64]	Yes	20	GLCM, NGLDM, GLZLM	LifeX	OS	20	Spearman correlation	17 (47.22%)
Lee H et al. [40]	Yes	nd	First-order, GLCM, GLRLM	Mazda	OS	19	Wilcoxon, Chi square	10 (27.78%)
Huang S Y et al. [77]	Yes	42	First-order, shape, texture	Pyradiomics	OS	10	Chi square	11 (30.56%)
Boughdad S et al. [65]	Yes	31	GLCM, GLRLM, NGLDM, GLZLM	LifeX	OS	6	Spearman correlation, Anova	6 (16.67%)
Molina-Garcia D et al. [73]	Yes	16	GLCM, GLRLM	Matlab	C	3	Kolmogorov–Smirnov, Mann–Whitney, ROC	17 (47.22%)
Yoon HJ et al. [78]	Yes	46	First-, second-order	CGITA	OS	37	Kolmogorov–Smirnov, Mann–Whitney, ROC	10 (27.78%)
Orlhac et al. [66]	Yes	9	GLCM, GLRLM, GLZLM	LifeX	OS	6	Wilcoxon	9 (25.00%)
Moscoso A et al. [59]	Yes	nd	First-order, GLCM, GLSZM	Matlab	C	10	Spearman correlation, Kruskal–Wallis	9 (25.00%)
Antunovic et al. [12]	Yes	17	First-order, shape, size	Matlab	C	12	Pearson correlation	8 (22.22%)
Cheng et al. [61]	Yes	3	Skewness, entropy, coarseness	Matlab	C	3	Chi square, ROC	9 (25.00%)
Ha et al. [75]	Yes	109	GLCM, GLRLM, NGLDM, GLZLM, GLSZM	CGITA	OS	109	Pearson correlation	8 (22.22%)
Yang et al. [67]	Yes	5	Pathological features	nd	nd	5	Spearman correlation	6 (16.67%)
Groheux et al. [55]	Yes	nd	GLCM	nd	nd	3	Wilcoxon test, Benjamini–Hochberg method	6 (16.67%)
Orlhac et al. [68]	Yes	6	Homogeneity, entropy, SRE, LRE, LGZE, HGZE	LifeX	OS	2	Wilcoxon	7 (19.44%)
Groheux et al. [32]	Yes	nd	nd	nd	nd	nd	Spearman correlation, ROC	13 (36.11%)
Yoon et al. [33]	Yes	9	Clinical PET-MRI/BSGI features	PMOD	C	9	Chi square, Kruskal–Wallis, Logistic regression analysis	6 (16.67%)
Hatt et al. [69]	Yes	15	GLCM	nd	nd	4	Spearman correlation, univariate Cox proportional hazards regression	5 (13.89%)
Soussan et al. [34]	Yes	31	First-order, GLCM, GLRLM	nd	nd	3	ROC, univariate logistic regression	9 (25.00%)
Orlhac et al. [70]	Yes	36	First-order, GLCM, GLRLM, NGLDM, GLZLM	nd	nd	7	Pearson correlation	16 (44.44%)
Willaime et al. [41]	Yes	28	First-order, GLCM, GLSZM, NGTDM	Matlab	C	8	Shapiro–Wilk, ICC	6 (16.67%)
Lemarignier et al. [74]	Yes	4	Entropy, homogeneity, contrast and energy	nd	nd	4	Spearman correlation	8 (22.22%)

BC: breast cancer; BSGI: breast-specific gamma imaging; C: commercial; GLCM: gray-level co-occurrence matrix; GLDM: gray-level difference matrix; GLRLM: gray-level run-length matrix; GLSZM: gray-level size zone matrix; GLZLM: gray-level zone-length matrix; FTs: features; HGZE: high gray-level zone emphasis; ICC: intra-class correlation; LASSO: least absolute shrinkage and selection operator; LGZE: low gray-level zone emphasis; LRE: long-run emphasis; nd: not defined; NGLDM: neighborhood gray-level different matrix; NGTDM: neighborhood gray-tone difference matrix; OS: open source; PCA: principal component analysis; ROC: receiving operating characteristics; RQS: radiomics quality score; SRE: short-run emphasis; Sw: software; TA: texture analysis.

**Table 3:** Summary of studies’ data mining (from newest to older).

Author	AI Area	AI Sw	Sw Class	Data-Mining Methods	Validated	Validation Test
Chen et al. [44]	ML	Pyton software and Pyradiomics module	OS	RF, SVM, SGD, KNN	Yes	5-fold cross-validation
Umutlu et al. [35]	DL	Matlab	C	SVM	Yes	5-fold cross-validation
Eifer et al. [43]	ML	Pyradiomics, Scikit-learn, TensorFlow libraries	OS	KNN and RF	Yes	5-fold cross-validation
Jo et al. [51]	nd	NA	NA	NA	No	NA
Cheng et al. [42]	ML	R-software	OS	Multivariable regression with the Akaike’s information criterion (AIC)	Yes	10-fold cross-validation
Castaldo et al. [62]	ML	R-software	OS	Additive logistic regression (LogitBoost), RF, LDA	Yes	3-fold cross-validation
Araz et al. [45]	ML	WEKA	OS	SVM, Hoeffding tree, J48, and MLP	Yes	10-fold cross-validation
Satoh et al. [56]	DL	Pytorch	OS	CNN based on Xception	No	NA
Takahashi et al. [63]	DL	Pytorch	OS	CNN based on Xception	No	NA
Moreau et al. [46]	DL	Python and Phytorch	OS	U-Net	Yes	5-fold cross-validation
Chen et al. [71]	ML	Python	OS	MLP, SVM, RF and XGBoost	Yes	3-fold cross-validation
Umutlu et al. [72]	ML	Matlab	C	SVM	Yes	5-fold cross-validation
Krajnc et al. [28]	ML	NA	NA	RF	Yes	100-fold MC-cross-validation
Weber et al. [53]	ML	Matlab	C	CNN	Yes	bootstrap Gauss test
Aide et al. [76]	ML	XLSTAT Software	C	RF	Yes	OOB
Li et al. [48]	DL	DCNN-based diagnosis method	IH	3D CNN	Yes	5-fold cross-validation
Song et al. [49]	ML	R	OS	XGBoost	Yes	NA
Choi et al. [37]	DL	CNN-based sofware	OS	CNN	Yes	3-fold cross-validation
Satoh et al. [60]	ML	scikit-learn and data mining framework in Pyton	OS	SVM	Yes	2-fold cross-validation
Li et al. [38]	ML	Scikit-learn, numpy, scipy and math packages in Pyton	OS/C	RF	Yes	10-fold cross-validation
Ou et al. [29]	ML	PYTHON and IBM SPSS	OS/C	LDA	Yes	10-fold cross-validation
Antunovic et al. [39]	ML	STATA/R	C/OS	Univariable and multivariable logistic regression	Yes	10-fold cross-validation
Aide et al. [64]	ML	XLSTAT Software	C	RF	Yes	OOB
Lee et al. [40]	ML	R	OS	Multivariable logistic regression	Yes	Cross-validation 10-fold, 5-fold, and leave-one-out methods
Huang et al. [77]	ML	Python	IH	SVM, RF, logistic regression	Yes	3-fold cross-validation

AI: artificial intelligence; BC: breast cancer; CNN: convolutional neural network; DL: deep learning; FTs: features; KNN: k-nearest neighbors; LDA: linear discriminant analysis; IH: in-house; ML: machine learning; MLP: multi-layer perceptron; NA: not applicable; nd: not defined; OOB: Out-Of-Bag; RF: random forest; SGD: stochastic gradient descent; SVM: support vector machine; Sw: software; TA: texture analysis; XGBoost: eXtreme Gradient Boosting.

## Data Availability

Not applicable.
